# Supplementary material for: The Spectrum of FANCM Protein Truncating Variants in European Breast Cancer Cases
Source: Cancers (Basel). 2020 Jan 26;12(2):292. doi: 10.3390/cancers12020292 (PMC7073216; doi:10.3390/cancers12020292)
Supplement: Supplementary file 1 [file cancers-12-00292-s001.pdf]

Supplementary Materials: The spectrum of *FANCM* protein truncating variants in European breast cancer cases

Gisella Figlioli, Anders Kvist, Emma Tham, Jana Soukupova, Petra Kleiblova, Taru A Muranen, Nadine Andrieu, Jacopo Azzollini, Judith Balmaña, Alicia Barroso, Javier Benítez, Birgitte Bertelsen, Ana Blanco, Bernardo Bonanni, Åke Borg, Joan Brunet, Daniele Calistri, Mariarosaria Calvello, Stepan Chvojka, Laura Cortesi, Esther Darder, Jesús Del Valle, Orland Diez, ENIGMA Consortium, Séverine Eon-Marchais, Florentia Fostira, GENESIS Study Collaborators, Francesca Gensini, Claude Houdayer, Marketa Janatova, Johanna I Kiiski, Irene Konstantopoulou, Katerina Kubelka-Sabit, Conxi Lázaro, Fabienne Lesueur, Siranoush Manoukian, Ruta Marcinkute, Ugnius Mickys, Virginie Moncoutier, SWE-BRCA Group, Aleksander Myszka, Tu Nguyen-Dumont, Finn Cilius Nielsen, Rimvydas Norvilas, Edith Olah, Ana Osorio , Laura Papi, Bernard Peissel, Ana Peixoto, Dijana Plaseska-Karanfilska, Timea Pócza, Maria Rossing, Vilius Rudaitis, Marta Santamariña, Catarina Santos, Snezhana Smichkoska, Melissa C Southey, Dominique Stoppa-Lyonnet, Manuel Teixeira, Therese Törngren, Angela Toss, Miguel Urioste, Ana Vega, Zdenka Vlckova, Drakoulis Yannoukakos, Valentina Zampiga, Zdenek Kleibl, Paolo Radice, Heli Nevanlinna, Hans Ehrencrona, Ramunas Janavicius, Paolo Peterlongo

Table S1. Center or Study contributing the 114 *FANCM* PTV probands. Some of these probands were previously described as indicated; sequencing details are provided.

Center/Study	Country	Number of Probands	Published Previously	SEQUENCING DESIGN
CZECANCA-AGEL, Novy Jicin	Czech Republic	1	No	Custom gene panel (NimbleGen, Roche; sequencing, Illumina)
CZECANCA-Charles University, Prague	Czech Republic	10	Yes [1]	Custom gene panel (NimbleGen, Roche; sequencing, Illumina)
CZECANCA-Gennet, Prague	Czech Republic	5	No	Custom gene panel (NimbleGen, Roche; sequencing, Illumina)
CZECANCA-GHC, Prague	Czech Republic	4	No	Custom gene panel (NimbleGen, Roche; sequencing, Illumina)
Copenhagen Breast Cancer Study	Denmark	4	No	Custom gene panel (NimbleGen, Roche; sequencing Illumina)
Helsinki Breast Cancer Study	Finland	4	Yes [2,3]	Whole exome sequencing (SureSelect, Agilent and NimbleGen, Roche; sequencing, Illumina); Custom gene panel (SureSelectXT, Agilent; sequencing, Illumina)
GENE SISters	France	8	Yes [4,5]	Custom gene panel (SureSelect, Agilent; sequencing, Illumina) and targeted Sanger sequencing
Institut Curie, Paris	France	7	No	Custom gene panel (SureSelect QXT, Agilent; sequencing, Illumina)
National Center for Scientific Research-Demokritos, Athens	Greece	6	Yes [6]	Gene panel (TruSight Cancer Panel; sequencing, Illumina)
Hungarian Breast and Ovarian Cancer Study	Hungary	2	No	Gene panel (TruSight Cancer Panel; sequencing, Illumina)
Fondazione IRCCS-Istituto Nazionale dei Tumori, Milan	Italy	2	Yes [7]	Whole exome sequencing (SureSelect Human All Exon Kit, Agilent; sequencing, Illumina)
Istituto Scientifico Romagnolo per lo Studio e la Cura dei Tumori, Meldola	Italy	1	Yes [8]	Gene panel (TruSight Cancer Panel; sequencing, Illumina)
Vilnius University Hospital Santaros Klinikos, Vilnius	Lithuania	7	No	Gene panel (TruSight Cancer Panel; sequencing, Illumina)

Macedonian Breast Cancer Study	Macedonia	2	No	Gene panel (TruSight Cancer Panel; sequencing, Illumina)
Wroclaw Medical University, Wrocław	Poland	3	Yes [9]	FANCM and RECQL amplicon-based massively parallel sequencing (Hi-Plex)
Portuguese Oncology Institute-Porto Breast Cancer Study	Portugal	3	No	Gene panel (TruSight Cancer Panel; sequencing, Illumina)
Catalan Institute of Oncology, Barcelona	Spain	5	Yes [10]	Whole exome sequencing (SureSelect Human All Exon, Agilent; sequencing, Illumina) and custom gene panel (SureSelect, Agilent or TruSight Cancer Panel, Illumina)
Fundación Pública Galega Medicina Xenómica, Santiago de Compostela	Spain	3	No	Custom gene panel (SureSelect, Agilent; sequencing, Illumina)
Spanish National Cancer Research Centre, Madrid	Spain	5	No	Onco-Gene SGKit gene panel, Sistemas Genomicos, Valencia.
Swedish Breast Cancer Study	Sweden	32	No	Custom gene panel (SureSelect, Agilent; sequencing, Illumina)

References

1 Lhota, F.; Zemankova, P.; Kleiblova, P.; Soukupova, J.; Vocka, M.; Stranecky, V.; Janatova, M.; Hartmannova, H.; Hodanova, K.; Kmoch, S.; et al. Hereditary truncating mutations of DNA repair and other genes in BRCA1/BRCA2/PALB2-negatively tested breast cancer patients. *Clin. Genet.* **2016**, *90*, 324–333, doi:10.1111/cge.12748.

2 Kiiski, J.I.; Pelttari, L.M.; Khan, S.; Freysteinsdottir, E.S.; Reynisdottir, I.; Hart, S.N.; Shimelis, H.; Vilske, S.; Kallioniemi, A.; Schleutker, J.; et al. Exome sequencing identifies FANCM as a susceptibility gene for triple-negative breast cancer. *Proc. Natl. Acad. Sci. USA* **2014**, *111*, 15172–15177, doi:10.1073/pnas.1407909111.

3. Kiiski, J.I.; Tervasmaki, A.; Pelttari, L.M.; Khan, S.; Mantere, T.; Pylkas, K.; Mannermaa, A.; Tengstrom, M.; Kvist, A.; Borg, A.; et al. FANCM mutation c.5791C > T is a risk factor for triple-negative breast cancer in the Finnish population. *Breast Cancer Res. Treat.* **2017**, *166*, 217–226, doi:10.1007/s10549-017-4388-0.

4. Girard, E.; Eon-Marchais, S.; Olaso, R.; Renault, A.L.; Damiola, F.; Dondon, M.G.; Barjhoux, L.; Goidin, D.; Meyer, V.; Le Gal, D.; et al. Familial breast cancer and DNA repair genes: Insights into known and novel susceptibility genes from the GENESIS study, and implications for multigene panel testing. *Int. J. Cancer* **2019**, *144*, 1962–1974, doi:10.1002/ijc.31921.

5. Peterlongo, P.; Catucci, I.; Colombo, M.; Caleca, L.; Mucaki, E.; Bogliolo, M.; Marin, M.; Damiola, F.; Bernard, L.; Pensotti, V.; et al. FANCM c.5791C>T nonsense mutation (rs144567652) induces exon skipping, affects DNA repair activity and is a familial breast cancer risk factor. *Hum. Mol. Genet.* **2015**, *24*, 5345–5355, doi:10.1093/hmg/ddv251.

6. Fostira, F.; Kostantopoulou, I.; Apostolou, P.; Papamentzelopoulou, M.S.; Papadimitriou, C.; Faliakou, E.; Christodoulou, C.; Boukovinas, I.; Razis, E.; Tryfonopoulos, D.; et al. One in three highly selected Greek patients with breast cancer carries a loss-of-function variant in a cancer susceptibility gene. *J. Med. Genet.* **2019**, 10.1136/jmedgenet-2019-106189, doi:10.1136/jmedgenet-2019-106189.

7. Gracia-Aznarez, F.J.; Fernandez, V.; Pita, G.; Peterlongo, P.; Dominguez, O.; de la Hoya, M.; Duran, M.; Osorio, A.; Moreno, L.; Gonzalez-Neira, A.; et al. Whole exome sequencing suggests much of non-BRCA1/BRCA2 familial breast cancer is due to moderate and low penetrance susceptibility alleles. *PLoS ONE* **2013**, *8*, e55681, doi:10.1371/journal.pone.0055681.

8. Tedaldi, G.; Tebaldi, M.; Zampiga, V.; Danesi, R.; Arcangeli, V.; Ravegnani, M.; Cangini, I.; Pirini, F.; Petracci, E.; Rocca, A.; et al. Multiple-gene panel analysis in a case series of 255 women with hereditary breast and ovarian cancer. *Oncotarget* **2017**, *8*, 47064–47075, doi:10.18632/oncotarget.16791.

9. Nguyen-Dumont, T.; Myszka, A.; Karpinski, P.; Sasiadek, M.M.; Akopyan, H.; Hammet, F.; Tsimiklis, H.; Park, D.J.; Pope, B.J.; Slezak, R.; et al. FANCM and RECQL genetic variants and breast cancer susceptibility: Relevance to South Poland and West Ukraine. *BMC Med. Genet.* **2018**, *19*, 12, doi:10.1186/s12881-018-0524-x.

10. Feliubadalo, L.; Tonda, R.; Gausachs, M.; Trotta, J.R.; Castellanos, E.; Lopez-Doriga, A.; Teule, A.; Tornero, E.; Del Valle, J.; Gel, B.; et al. Benchmarking of Whole Exome Sequencing and Ad Hoc Designed Panels for Genetic Testing of Hereditary Cancer. *Sci. Rep.* **2017**, *7*, 37984, doi:10.1038/srep37984.

© 2020 by the authors. Licensee MDPI, Basel, Switzerland. This article is an open access article distributed under the terms and conditions of the Creative Commons Attribution (CC BY) license (http://creativecommons.org/licenses/by/4.0/).
